# Reflex Auriculo-Cardiac (RAC) Induced by Auricular Laser and Needle Acupuncture: New Case Results Using a Smartphone

**DOI:** 10.3390/life13030853

**Published:** 2023-03-22

**Authors:** Ying-Ling Chen, Kun-Chan Lan, Mark C. Hou, He-Hsi Tsai, Gerhard Litscher

**Affiliations:** 1 School of Chinese Medicine, China Medical University, Taichung 404, Taiwan; lingcmu@cmu.edu.tw (Y.-L.C.);; 2 Department of Chinese Medicine, China Medical University Hospital, Taichung 404, Taiwan; 3 Department of Computer Science and Information Engineering (CSIE), National Cheng Kung University, Tainan 701, Taiwan; 4 Department of Chinese Medicine, Changhua Christian Hospital, Changhua 500, Taiwan; 5 Department of Beauty Science, Chien-Kuo Technology University, Changhua 500, Taiwan; 6 Department of Traditional Chinese Medicine, Taipei City Hospital Linsen Chinese Medicine and Kunming Branch, Taipei 104, Taiwan; 7 President of ISLA (International Society for Medical Laser Applications), Research Unit of Biomedical Engineering in Anesthesia and Intensive Care Medicine, Research Unit for Complementary and Integrative Laser Medicine, Traditional Chinese Medicine (TCM) Research Center Graz, Department of Anesthesiology and Intensive Care Medicine, Medical University of Graz, Auenbruggerplatz 39, 8036 Graz, Austria

**Keywords:** reflex auriculo-cardiac (RAC), dynamic pulse reaction, Nogier reflex, vascular autonomic signal, auricular acupuncture, photoplethysmography, smartphone, auricular medicine, evidence-based complementary medicine

## Abstract

The reflex auriculo-cardiac (RAC), dynamic pulse reaction (Nogier reflex), or vascular autonomic signal was proposed by Nogier. It refers to the pulse changes that can occur in the radial artery immediately after auricular acupuncture is performed. RAC is helpful for the clinical practice of auricular acupuncture, but there is a lack of objective verification methods. Photoplethysmography (PPG) has been used to objectively calculate radial artery blood flow. This study used PPG via a smartphone to measure RAC induced by auricular acupuncture. Thirty subjects without major diseases were recruited to receive traditional needle and laser acupuncture. The Shen Men ear point and control points were stimulated for 20 s. PPG was continuously measured during the acupuncture. The PPG data were tested for differences with a paired *t*-test. The results showed that there were no statistical differences in the frequency and amplitude of PPG obtained before and after acupuncture, either with a traditional needle or laser acupuncture. However, interestingly, it was found that one patient with insomnia, one patient with viral respiratory symptoms, and two menstruating females exhibited changes in PPG within five seconds of needle placement. We hypothesized that RAC might be induced by auricular acupuncture and could be quantified by PPG, even among subjects suffering from mild diseases; however, auricular acupuncture might not induce a measurable RAC in totally healthy subjects.

## 1. Introduction

Paul Nogier found that there are acupuncture points and/or areas on the ear. When subjected to physical stimulation or illness, these small points become sensitive to pain and can be detected by electronic equipment [1,2]. He also drew a map of the areas of the ear corresponding with various parts of the human body. In addition to diagnosis, acupuncture, massage, infrared rays, etc., can also be used for treatment [3,4,5]. Nogier discovered that applying light pressure to the ear also altered the pulse quality of the radial artery, leading to the reflex auriculo-cardiac (RAC) theory. The RAC, dynamic pulse reaction, Nogier reflex, or vascular autonomic signal can cause a vascular reflex through skin stimulation. The source of stimulation can be mechanical, acoustical, or optical in nature. By monitoring pulse changes in the radial artery, skin irritation to the organism can also be assessed. Experienced physicians can detect RAC by sensing changes in the quality of the radial pulse. RAC occurs normally 1 to 3 heart beats after stimulation and can be maintained for 8 to 15 beats. Pulse changes are dominated by an increased amplitude [6,7,8]. To quantify RAC, Litscher et al. used high-resolution imaging techniques to record pulse skin surface changes and clearly observed small changes in pulse on the skin surface [9]. Moser et al. found that there are two types of RAC, fast and slow, and speculated that the rapid changes in heart rate were due to vagal responses, whereas the slower changes observed in pulse waves were due to sympathetic nervous system responses [10]. Litscher et al. further proposed that RAC detection needed to include the concept of a multi-modal research approach [11].

On the other hand, the research on RAC is quite related to vagus nerve stimulation (VNS), which is an effective method for treating patients with intractable epilepsy and depression. However, because it is an invasive treatment, it has certain severe side effects [12]. Thus, it has been developed into transcutaneous vagus nerve stimulation (tVNS), which achieves similar effects through a transcutaneous approach and non-invasive skin stimulation [13]. As the vagus nerve is connected to the external auditory canal and the skin of the ear, there is not only RAC, but also an ear–cough reflex and an ear gag reflex. It shows that many different reflexes can occur from the ear through the vagus nerve in the body, and this is worth exploring in depth [14]. Therefore, as with the principle of tVNS, we may have the same effect without serious side effects by performing acupuncture, massage, or sound/light stimulation on the ear through the RAC theory, with a good treatment effect for patients. Evidence-based research on this topic can only be obtained when appropriate instruments to detect and analyze RAC are developed.

Photoplethysmography (PPG) is a plethysmography method that uses optics to measure organ volume. PPG uses light to illuminate the skin for measurement, and calculates normalized data by detecting the voltage change caused by the amount of light reflected or transmitted by the light-sensing element. Different media have different absorbances of light; when the blood flow under the skin changes, the absorbance of light will also change, resulting in changes in the amount of reflected light. PPG records these signals and plots the volume change of the blood flow; thus, changes in the subcutaneous blood can be obtained. The common clinical oximeter applies this principle [15,16]. PPG is a non-invasive measurement, which has the advantages of a small size, being user-friendly, and not being limited to a certain body part. It has been widely used in cardiovascular disease and hemodynamic research [17,18]. Although PPG signals and pulse waves are two different signals, they are both affected by cardiac rhythm movement and there is a definite correlation between them [19]. Therefore, the characteristics of PPG can be used for the detection of RAC.

As smartphones are very popular, many wearable devices and physiological parameter measurements are often combined with smartphones. For example, a smartphone camera is used to collect video from a subject’s fingertip, which is then converted into a PPG signal. High-frequency noise, optical noise, and motion interference are removed from the original PPG signal. These preprocessed signals are then used to extract signal features that can measure blood glucose [20]. A study was conducted with 217 participants in Cuenca, Ecuador. The design of a novel non-invasive blood glucose estimation system utilizing wristband PPG signals and physiological parameters was presented. The correlation coefficient between the results and the standard method was 0.99. It showed that the predicted glucose value was clinically usable [21]. A similar smartphone, combined with PPG under the calculation of artificial intelligence, was used to estimate the heart rate (HR) using PPG in fingertip video captured by the smartphone camera. It was based on tracking subtle changes in skin color due to cardiovascular activity. These color changes can be detected by digital cameras. The color intensity of the skin pixels was used and a filter was applied to remove noise and retain only pulses of interest. The extracted signal was fed to a convolutional recurrent neural network that output an estimated HR. The obtained results were compared with the real HR obtained using a contact PPG sensor [22]. Using smartphones to measure PPG is not only convenient, but also has great potential for uploading data to the cloud to build a database and subsequent analysis of big data. The Brno University of Technology smartphone PPG database (BUT PPG) was used to assess PPG quality and estimate HRs. The data consisted of 48 PPG recordings of 10s and the associated ECG signal was used to determine the reference HR. Data were collected from 12 subjects between the ages of 21 and 61. The recording time was 3 months. PPG data were collected using a Xiaomi Mi9 smartphone. Each PPG signal contained quality annotations and a reference HR [23].

In this study, in order to confirm that PPG is able to successfully measure changes in RAC, PPG was applied to 30 subjects. It was found that waveform changes could be found via PPG in the first second after the start of ear acupuncture in several patients [24]. A PPG evaluation was performed on subjects before and after ear acupuncture stimulation through laser acupuncture and traditional steel needle acupuncture in order to quantify possible RAC responses of the radial artery induced by acupuncture from the two modalities.

This article is organized as follows. In the first section, by describing the background of the study, we introduce the concepts of RAC and PPG. We then introduce our method in the next section, including the principle of volunteer recruitment, the case study process of acupuncture and PPG measurement, and the statistical methods for evaluation. In Section 3, we demonstrate the experimental case report results. In the fourth section, we discuss important arguments and propose future work. Finally, we make a conclusion of the study.

## 2. Materials and Methods

### 2.1. Recruitment of Subjects

Thirty healthy subjects were recruited for PPG measurement during ear acupuncture. According to Gay et al., at least 30 samples are needed for a difference to be detected [25]. The inclusion criterion was adults aged 20–50. Exclusions were pregnant women or those with hypertension, cardiovascular disease, diabetes, and other critical diseases. All subjects signed an informed consent form. This case report study was approved by the Institutional Review Board of Changhua Christian Hospital (no. 191215).

### 2.2. Application of Ear Acupuncture and Measuring Instruments

**Figure 1:**
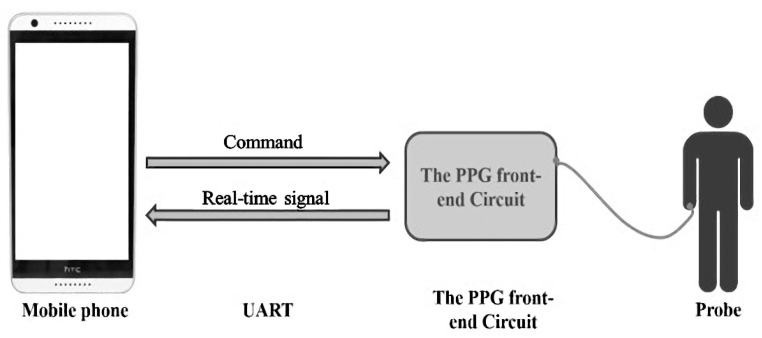
Photoplethysmography (PPG) signal diagram.

**Figure 2:**
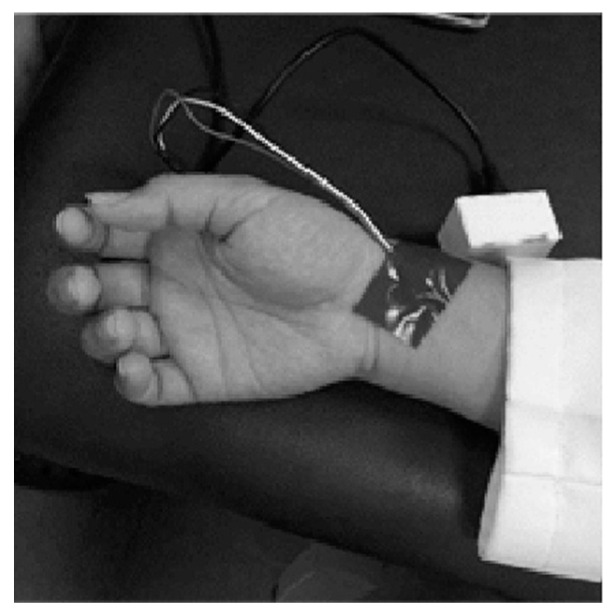
PPG probe on radial artery of the wrist.

A 0.5 inch 32 gauge stainless steel needle was used for ear acupuncture stimulation. Laser acupuncture was performed with an RJ Laser instrument (Reimers & Janssen GmbH, Germany) with a power level of 500 mJ, a frequency of 1168 Hz (Nogier), and an infrared wavelength of 810 nm. This frequency is mainly used for bones, muscles, joints, and extremities. It is a commonly used treatment option in clinical practice. The PPG-measuring instrument and software used in this research were developed by the Information Engineering Institute of National Cheng Kung University [17]. The hardware used a PPG optical probe (Arduino Pulse Sensor) to capture the radial artery signal (sampling every 0.04 s), and transmitted it to a mobile phone through the signal line to record and analyze the PPG data, which made the PPG measurement accessible (Figure 1 and Figure 2). The PPG accuracy, defined as the proportion of PPG measurements within 100 ms of electrocardiographic measurements, was ~95%. First, we located the wrist pulse position by locating the radial styloid process, and attached the PPG probe onto the radial styloid process with opaque adhesive tape. It was confirmed that the tape was tightly pasted and no light could enter, which would have affected the imaging function of the probe. After the measurement, the data were uploaded to a cloud database for further analysis [17].

### 2.3. Measurement Process

**Figure 3:**
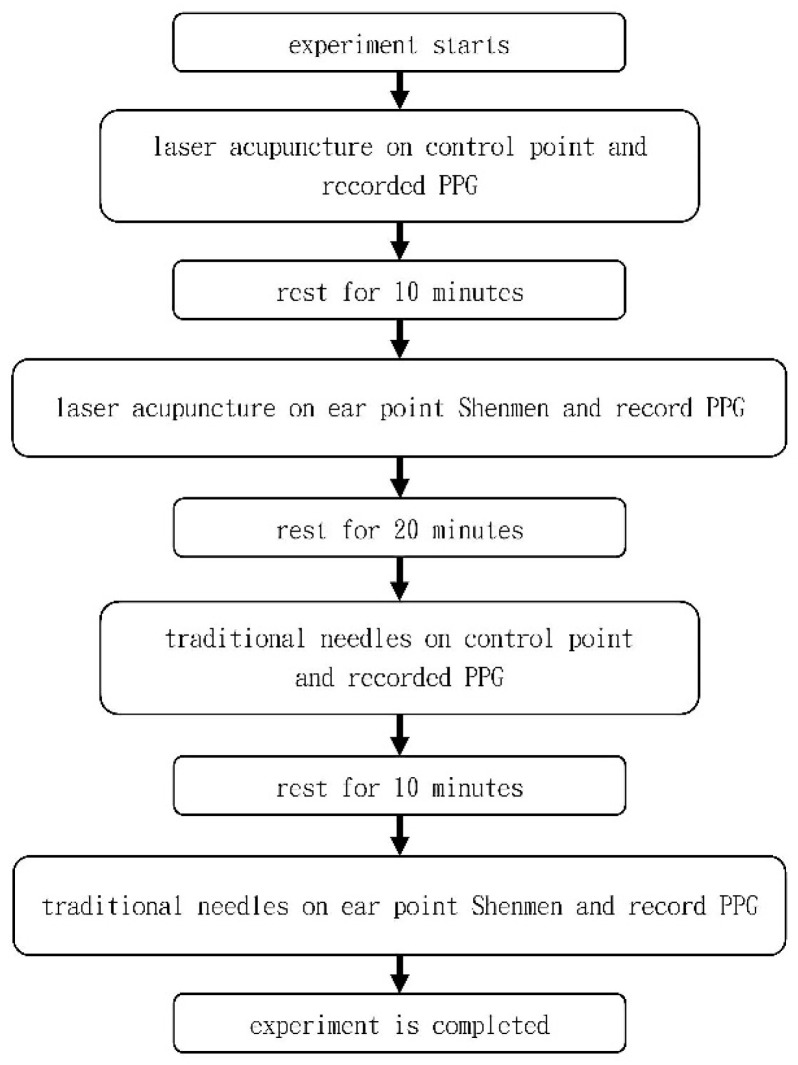
PPG measurement procedure.

The subjects received laser acupuncture to stimulate the control acupoints (behind the ears; no acupuncture point location) and PPG was recorded. After the session and a rest period of 10 min, they received laser acupuncture to stimulate the Shen Men ear point and PPG was recorded again. After the session and another 20 min rest, the subjects received manual needle acupuncture with stainless steel needles at the control point. After a 10 min rest, they then received acupuncture at the Shen Men ear point with stainless steel needles. Again, PPG was recorded (Figure 3). The Shen Men ear point is located at the back and top of the upper triangular fossa of the ear. For comparison, the control point was at the mastoid process of the temporal bone and at the same level as the Shen Men ear point. This position belongs to the great auricular nerve control, the same as the Shen Men ear point [5,6,7]. PPG was measured on the right wrist. There was a five second wait for the PPG signal to stabilize; this was the preparatory phase. From 6 to 25 s was the A phase, and there was no ear acupuncture stimulation for 20 s. From 26 to 45 s was the B period, and acupuncture was performed for 20 s from 26 s onwards. During the research process, in order to reduce the interaction between the stainless steel needle and the laser, an interval of 20 min was used as a washout for the effect. In addition, laser stimulation was performed first, followed by traditional stainless steel needle stimulation, mainly to avoid the pain caused by stainless steel needles. Therefore, the laser was performed first, because most people usually do not have obvious neurological reactions when receiving laser stimulation.

In this study, the Shen Men ear point was selected as the auricular acupuncture stimulation site. The reason for this was as follows. First, the Shen Men ear point is a very commonly used point in clinical work. Most researchers are familiar with it. Second, the Shen Men ear point has the effect of calming the nerves and helping sleep. Its working belongs to the nervous system, and the nature of the autonomic nervous system is relatively close to that of RAC/vagus nerve. Finally, the Shen Men ear point is less directly related to the cardiovascular system, which avoids interfering with the possible interrelationship between the ear and the cardiovascular system in RAC.

### 2.4. Evaluation

The PPG data of 30 subjects were analyzed, and the PPG peak interval (RR interval) and the highest peak value (amplitude) of the main peak were calculated using MATLAB (MathWorks, Natick, MA, USA). The data of PPG could be drawn into a graph similar to an electrocardiogram along with the recording time. Each heartbeat made a pulse to beat, and a corresponding waveform could be displayed on the PPG graph. The frequency of PPG could be obtained by calculating the time between the peak intervals (RR interval). The amplitude of PPG could be obtained by calculating the average height of the peak. In addition, the PPG data were standardized and the unit was expressed in arbitrary units (a.u.). The signal period was calculated in micro seconds. In addition, the mean and standard deviation (SD) were calculated. We tested whether there was a significant difference in RR interval and amplitude changes before and after acupuncture on the Shen Men ear point and the control points with steel needles and laser stimulation. If there was a difference, we compared their averages to see which one had the more significant change. At the same time, the PPG of the stainless steel needle and the laser was compared to analyze the difference as well. This study used SPSS 12.0 statistical software for the analysis. As the samples were dependent samples, a paired *t*-test was used to test for differences.

## 3. Case Results

A total of 30 subjects were recruited for this study. Among them were 8 men and 22 women. As there was no current literature showing if RAC was significantly affected by gender, it should not have affected the results, although the proportion of males and females included as subjects was uneven. All subjects signed a consent form. Table 1 shows their basic information (demographic data).

There was no statistical difference in PPG before and after puncturing the Shen Men ear point and control points with steel needles (Table 2).

There was also no statistical difference in PPG before and after laser acupuncture stimulation of the Shen Men ear point and the control point (Table 3).

Comparing the PPG data of the Shen Men ear point and the control point before and after acupuncture with laser or steel needle stimulation also did not show a statistically significant difference. In addition, when comparing the laser and the steel needle, the stimulation with the steel needle caused frequency changes in both the Shen Men ear acupuncture point and the control points (*p*-values of 0.039 and 0.018, respectively), but this was not so with laser acupuncture.

**Figure 4:**
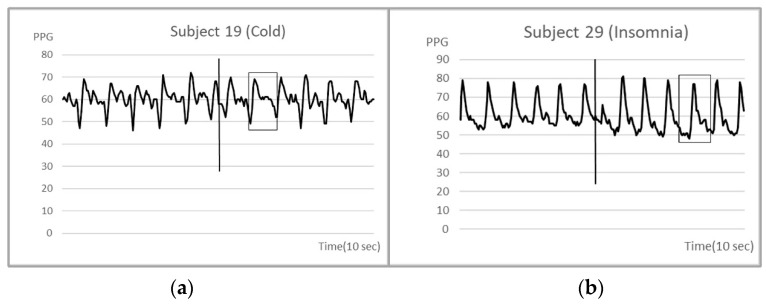
(**a**) The frequency of subject number 19 with a cold slowed down after acupuncture; (**b**) the amplitude increased after acupuncture on subject number 29 with insomnia. The vertical line in the figure indicates the start of the acupuncture and the rectangle shows the maximum of alteration.

**Figure 5:**
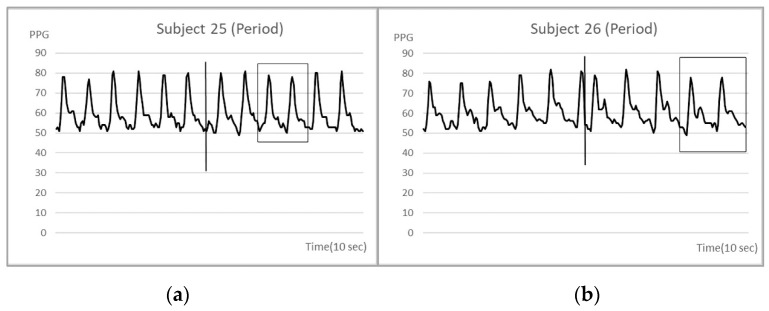
(**a**) Subject number 25 had a decrease in amplitude after acupuncture during her menstruation period; (**b**) subject number 26 has a decrease in amplitude after acupuncture during her menstruation period as well. The vertical line in the figure indicates the start of the acupuncture and the rectangle shows the maximum of alteration.

We further examined the waveform changes of PPG over five seconds before and after acupuncture, and found that there were four subjects with PPG frequency or amplitude changes. These four subjects had insomnia, cold problems, and menstruation. The five second PPGs of the four subjects before and after acupuncture are shown in Figure 4 and Figure 5, respectively. It could be seen that PPG slightly changed immediately after acupuncture.

## 4. Discussion

This case report study sought to confirm acupuncture-induced RAC using PPG. However, judging from the results, acupuncture stimulation of the Shen Men ear point did not lead to statistically significant changes in PPG. This may be due to the fact that the subjects in this study were healthy volunteers. Only a few of them had mild ailments (insomnia, etc.). It was also possible that the PPG sampling time was too long to detect differences. We did find changes in PPG following acupuncture in two people (with insomnia and an acute viral upper respiratory illness, respectively) and in two female subjects during menstruation. These PPG changes were very subtle, occurred within five seconds after acupuncture, and only occurred within one to two PPG cycles. The induction of RAC may require that the subject has a pathological condition. This study was the first to use PPG to measure the characteristics of RAC and may provide an important reference for future RAC research.

PPG has advantages in measuring pulse changes. Temko et al. applied the method to measure heart rhythm during exercise [26]. In this study, PPG was combined with a smartphone. PPG data can be uploaded to the cloud for storage in real-time, so this is a portable solution that can be flexibly used in clinical and home healthcare. Combined with cloud computing, it can be developed for telemedicine in the future.

According to Moser et al.’s study using electrocardiogram, pulse wave, and respiratory synchronization analyses, RAC first produces a vagus nerve-controlled fast reflex after stimulation to slow the heartbeat (with a delay of one to five seconds), and then a sympathetic nerve-controlled slow reflex to slow the heartbeat. Rapid changes in heart rate are vagal responses to small stimuli, whereas slower changes observed in pulse waves originate from sympathetic nervous system responses [10]. The source of the PPG signal is the change in peripheral blood circulation, which is directly affected by the heartbeat. Therefore, it is reasonable to speculate that PPG should also have this response, but the data did not show that. This was consistent with the observation by Moser et al. that infrared lasers had no effect on heart rhythm amplitude. In order to understand whether the PPG change caused by ear acupuncture was due to pain, laser acupuncture was added in addition to traditional manual steel needle acupuncture. Usichenko et al. believed that the analgesic effect of auricular points was transmitted by stimulating the vagus nerve [27]. He et al. stated that the auricular acupuncture function was the stimulation of the auricular vagus nerve to change the autonomic nervous system and central nervous system [28]. Therefore, the addition of laser acupuncture should have been able to distinguish the effect from that of traditional manual needle acupuncture; however, the results showed no difference. The reason remains unclear.

There was no difference in PPG between the stimulation of the Shen Men ear point and the control points. According to the research of He et al. and Ueno et al., the pain sensation at the location of the Shen Men ear point is mainly received by the auriculotemporal nerve, the lesser occipital nerve, and a part of the vagus nerve auricular branch [28,29]. However, Usichenko et al. believed that this was the innervation of the vagus auricular branch and the greater auricular nerve [27]. The control point behind the ear may be innervated by the greater auricular nerve and the lesser occipital nerve. We assumed that the control belonged to the same dermatome as the Shen Men ear point. It should have a similar acupuncture response, but the exact neuroanatomical divisions here are cognitively divergent and the individual differences are great, adding more variables. The selection of control points for acupuncture research is still an unresolved issue [30].

In a previous study, we found that pulse changes could be observed using a smartphone. Moreover, we found that several PPG measurement results using smartphones were consistent with the RAC theory [24]. Drawing on that, we performed a large-scale experiment; surprisingly, we did not detect pulse changes in all subjects. Therefore, we conducted an analysis, and found that those with pulse changes had health problems or were menstruating. However, these subjects comprised only a small number, which could not be detected from a statistical test. Therefore, we developed the hypothesis that RAC can only be observed in symptomatic people; thus, this could be useful as a diagnostic tool.

There are problems with the use of words between different languages in the discussion of the term reflex auriculo-cardiac (RAC). For example, we once searched for a commonly used herb (*Angelica sinensis*) using three different terms—namely, Dong-quai, Dang guim and Dang qui—to find all available documents. Especially in today’s extensive use of electronic/online databases such as PubMed and Google Scholar, we must pay attention to this problem. As RAC was first used by Nogier, subsequent researchers often use this term. We think that for these predecessors, this term should be continued; of course, it can be changed to auriculo-cardiac reflex (ACR) for the convenience of English readers, as long as the researcher can understand that it is only a synonym. Earlier, Nogier discovered the phenomenon of RAC through manual manipulation. Nogier performed acupuncture on a point of a patient’s ear with one hand and pressed the pulse of the patient’s radial pulse with the other hand, feeling the subtle changes in the patient’s pulse and dimension from 1–3 heartbeats after acupuncture. He obtained confirmation of the existence of RAC from such a dedicated operation. The researchers behind him also took great efforts to use expensive and huge instruments as well as microscopic techniques to observe the tiny skin vibrations caused by the pulse of the skin of the hand during acupuncture. The present research used the latest technology, which combined smartphones and PPG measurements to find traces of RAC; it has also passed through many difficult tests. We sincerely hope that through the efforts of generations of researchers, we can unravel the mystery of RAC and the benefits to human health.

The next step in RAC research could be undertaken from two perspectives. The first is to confirm that the RAC found in this study only occurs in non-healthy individuals. Appropriate diseases or specific physical conditions should be selected such as those found in this study with insomnia or menstruating women. Large PPG samples should be collected and the PPG acquisition time shortened to ten seconds before and after acupuncture. It may then be possible to obtain enough data to confirm that the objective signal change from RAC is statistically significant. Other different diseases should then be selected to measure RAC in the same way to know if RAC represented by different diseases is different and if it can be used as a reference for diagnosis or treatment. This would provide an important basis for the development of RAC/PPG, and make it a very good adjunct to modern medicine. The second aspect is the optimization of PPG measurements for smartphones. A PPG probe should be integrated into smartphones or wireless transmission adopted to increase integration and stability, avoid measurement errors, and reduce noise interference. Adding 5G transmission would make the data response faster and more accurate. Cross-field cooperation between information technology and medicine will provide more convenient, stable, and real-time data collection and analysis, and with the help of big data calculation, RAC can be more conveniently used as an important part of mobile health devices. The health knowledge of acupuncture and complementary medicine should be further combined, promoted to preventive medicine, and become an important part of the health industry. At the moment, it is not possible to compare PPG changes with other certified equipment because there are no standard methods or comparable results concerning RAC measurements available.

For future studies, it would be a good recommendation to examine one group of individuals with known cardiovascular problems, which could be verified by an evaluation of an RAC auriculotherapy assessment, rather than evaluate healthy subjects alone.

## 5. Conclusions

In this case presentation study, radial artery blood flow was measured in thirty volunteers before and after they received conventional and laser auricular acupuncture stimulation via a smartphone connected to a PPG probe, and PPG data were obtained. An attempt was made to confirm the existence of RAC. RAC relates to the pulse changes that can occur in the radial artery immediately after ear acupuncture is performed by comparing changes in PPG as objective evidence of RAC.

In conclusion, acupuncture stimulation did not produce a statistically demonstrable difference in RAC in healthy subjects when PPG was used as the evaluation method. However, acupuncture stimulation in symptomatic subjects induced PPG-visible RAC changes within five seconds. PPG coupled with a smartphone could be an excellent measurement tool and is worth considering.

## Figures and Tables

**Table 1:** Demographic data (mean ± SD).

N = 30	Male	Female	Total
	8	22	30
Age (years)	30.25 ± 3.01	31.59 ± 6.10	31.23 ± 5.43
Height (cm)	172.88 ± 4.12	159.68 ± 3.87	163.20 ± 7.08
Weight (cm)	73.00 ± 8.93	53.50 ± 5.17	58.70 ± 10.75

**Table 2:** Comparison of PPG between Shen Men ear point and control point (traditional needle stimulation; a.u.: arbitrary units).

	Shen Men Ear Point			Control Point		
	A	B	*p*-Value	A	B	*p*-Value
RRI ^1^ (ms)	0.83 ± 0.10	0.84 ± 0.10	0.535	0.83 ± 0.10	0.84 ± 0.10	0.256
Amplitude (a.u.)	60.11 ± 2.11	60.07 ± 1.81	0.824	60.11 ± 2.11	60.07 ± 1.81	0.561

^1^ RRI: RR interval.

**Table 3:** PPG by laser acupuncture on Shen Men ear point and control point.

	Shen Men Ear Point			Control Point		
	A	B	*p*-Value	A	B	*p*-Value
RRI (ms)	0.85 ± 0.09	0.86 ± 0.10	0.074	0.82 ± 0.09	0.82 ± 0.09	0.899
Amplitude (a.u.)	60.14 ± 1.03	60.12 ± 0.91	0.827	60.36 ± 1.32	60.51 ± 1.18	0.249

## Data Availability

We have provided our data on Google Drive for reference: https://drive.google.com/drive/folders/1Tf52PDHO8GPIHtM6mhTu0TARXmxZxKwr?usp=sharing (accessed on 20 March 2023).
